# The Deubiquitylase USP4 Interacts with the Water Channel AQP2 to Modulate Its Apical Membrane Accumulation and Cellular Abundance

**DOI:** 10.3390/cells8030265

**Published:** 2019-03-21

**Authors:** Sathish K. Murali, Takwa S. Aroankins, Hanne B. Moeller, Robert A. Fenton

**Affiliations:** InterPrET Center, Department of Biomedicine, Aarhus University, Aarhus, DK-8000 Aarhus C, Denmark; sathish@biomed.au.dk (S.K.M.); takwa@biomed.au.dk (T.S.A.); hbmo@ana.au.dk (H.B.M.)

**Keywords:** water transport, NDI, aquaporin, ubiquitylation, post-translational modification

## Abstract

Aquaporin 2 (AQP2) mediates the osmotic water permeability of the kidney collecting duct in response to arginine vasopressin (VP) and is essential for body water homeostasis. VP effects on AQP2 occur via long-term alterations in AQP2 abundance and short-term changes in AQP2 localization. Several of the effects of VP on AQP2 are dependent on AQP2 phosphorylation and ubiquitylation; post-translational modifications (PTM) that modulate AQP2 subcellular distribution and function. Although several protein kinases, phosphatases, and ubiquitin E3 ligases have been implicated in AQP2 PTM, how AQP2 is deubiquitylated or the role of deubiquitylases (DUBS) in AQP2 function is unknown. Here, we report a novel role of the ubiquitin-specific protease USP4 in modulating AQP2 function. USP4 co-localized with AQP2 in the mouse kidney, and in mpkCCD14 cells USP4 and AQP2 abundance are increased by VP. AQP2 and USP4 co-immunoprecipitated from mpkCCD14 cells and mouse kidney, and in vitro, USP4 can deubiquitylate AQP2. In mpkCCD14 cells, shRNA mediated knockdown of USP4 decreased AQP2 protein abundance, whereas no changes in AQP2 mRNA levels or VP-induced cAMP production were detected. VP-induced AQP2 membrane accumulation in knockdown cells was significantly reduced, which was associated with higher levels of ubiquitylated AQP2. AQP2 protein half-life was also significantly reduced in USP4 knockdown cells. Taken together, the data suggest that USP4 is a key regulator of AQP2 deubiquitylation and that loss of USP4 leads to increased AQP2 ubiquitylation, decreased AQP2 levels, and decreased cell surface AQP2 accumulation upon VP treatment. These studies have implications for understanding body water homeostasis.

## 1. Introduction

Aquaporin 2 (AQP2) is the arginine vasopressin (VP) regulated water channel of the kidney collecting duct (CD). A major role of VP in regulating body water homeostasis involves binding of VP to the vasopressin type 2 receptor (V2R) in collecting duct principal cells, which initiates a complex intracellular signaling response leading to apical plasma membrane targeting of AQP2 [1,2]. Various post-translational modifications of AQP2 in its intracellular carboxyl-terminal tail play a vital role in determining its subcellular distribution and function. For instance, phosphorylation of AQP2 at Ser-256, Ser-261, Ser-264, and Ser-269 (Thr-269 in human) plays various roles in modulating AQP2 membrane accumulation, rate of degradation, or AQP2 recycling pathways [3,4]. In contrast, AQP2 is polyubiquitylated with K63 linked chains at Lys270, which increases AQP2 endocytosis and lysosomal degradation [5]. Although AQP2 can be phosphorylated and ubiquitylated simultaneously, phosphorylation of AQP2 is able to override the internalization signal of ubiquitin [3].

Ubiquitylation is an enzymatic process involving three different class of enzymes, namely ubiquitin-activating enzymes (E1), ubiquitin-conjugating enzymes (E2), and ubiquitin-ligating enzymes (E3) [6]. Several E3 ubiquitin ligases have been implicated in regulation of AQP2. For example, BRE1B, CUL5, and Nedd4-1 are altered in abundance by VP, suggesting a role in the regulation of water homeostasis [7]. Nedd4-1 and Nedd4-2 were further demonstrated to mediate ubiquitylation and degradation of AQP2 via the NEDD4 family interacting proteins NDFIP1/2 [8]. In addition, we recently determined that the c-terminal of Hsp70 interacting protein (CHIP) ubiquitylates AQP2 and plays a dual role in AQP2 degradation via ERAD or the lysosome and ultimately water balance [9].

In contrast, how AQP2 is deubiquitylated, or the role of deubiquitylases (DUBS) in AQP2 function is unknown. The human genome encodes for approximately 95 DUBS, which are classified into cysteine protease DUBS and metalloprotease DUBS [10]. Cysteine DUBS are further sub-classified based on their ubiquitin-protease domains into ubiquitin-specific proteases (USP), ubiquitin C-terminal hydrolases (UCH), Machado–Joseph disease proteases (MJD), and Otubain domain-containing proteases (OTU) [10]. In a recent proteomics screen, we determined that several of these classes of DUBS, including USP4, USP5, USP7, OTUB1, and ATXN3, were enriched in mouse cortical collecting duct cells (mpkCCD14) suggesting that they may play a role in removing the polyubiquitin chain from AQP2 [9].

As USPs display high substrate specificity [11], and USP4 is involved in the Wnt/β-catenin [12] and NF-κB signaling pathways [13] that regulate AQP2 expression [14,15], we hypothesized that USP4 has a role in modulating AQP2 function. We demonstrate that USP4 is abundantly expressed in mouse kidney and mpkCCD14 cells, where it co-localizes and interacts with AQP2. USP4 can reduce AQP2 ubiquitylation levels, and as such plays a role in AQP2 membrane accumulation and modulating AQP2 protein half-life. Taken together, we highlight for the first time specific regulation of AQP2 function by a DUB. Our studies have implications for general understanding of body water homeostasis. 

## 2. Materials and Methods

### 2.1. Antibodies and Chemicals

Antibodies used were against; USP4 (2651, Cell Signaling), AQP2 [16], Ser256 phosphorylated AQP2 [17], proteasome 20s (ab3325, Abcam), α-ENaC [18], actin (A2228, Sigma), AQP4 (AQP-004, Alomone, Jerusalem, Israel), V2R [19], Na-K-ATPase α1 subunit [20], H+-ATPase B1 subunit [21] and ubiquitin (P4D1, Cell Signaling). DUB inhibitor PR619 (Sigma), phosphatase inhibitor, and protease inhibitor (PhosSTOP and cOmplete mini tablets, Roche Diagnostics A/S) were added to all the isolation and wash buffers used in immunoprecipitation and biotinylation experiments. 

### 2.2. Cell Culture Conditions

mpkCCD14 cells were cultured in complete DMEM/F12 media as described earlier [22] on semi-permeable supports (Corning, Kennebunk, ME, USA). Cells were grown until formation of a confluent monolayer and transepithelial resistance (TER) was >5 kOhm/cm^2^. To induce AQP2 expression, serum-free complete DMEM/F12 media with 10^−9^ M desmopressin (dDAVP) was added to the basolateral compartment for 4 days. On the day of the experiment, cells were washed twice with DMEM/F12 and incubated in DMEM/F12 for 2 h at 37 °C. For experiments involving acute dDAVP treatment, cells were incubated in DMEM/F12 containing either vehicle or dDAVP (10^−9^ M) for 20 min at 37 °C. For experiments involving forskolin treatment, cells were washed twice with DMEM/F12, and incubated in DMEM/F12 media containing either vehicle or forskolin (25 μM) for 15 min at 37 °C. After incubation, cells were washed with DMEM/F12 media and proteins were extracted using Laemmli sample buffer containing 15 mg/mL DTT. 

### 2.3. shRNA Mediated USP4 Knockdown in mpkCCD14 Cells

For shRNA mediated USP4 knockdown, mpkCCD14 cells were grown on 12-well plastic plates without antibiotics until 80% confluent. Cells were treated with hexadimethrine bromide (8 μg/mL) for 30 min. Subsequently, cells were incubated with MISSION lentiviral transduction particles against USP4 (TRCN0000030743 (shRNA 1) and TRCN0000295778 (shRNA 2), (SHCLNV-NM_011678, Sigma) or control lentiviral particles (SCH 002V, Sigma). After 24 h, cells were grown in media containing 2 μg/mL Puromycin. Several clonal lines were isolated and individually characterized based on morphology, growth characteristics, Usp4 mRNA, and protein expression. 

### 2.4. Mouse Tissue Isolation and Immunolabeling

All animal protocols comply with the European Union guidelines for the use of experimental animals and were performed in accordance to licenses issued by the Danish Ministry of Justice. C57bl6/J mice were euthanized by cervical dislocation. Kidney cortex, heart, and spleen were removed and homogenized in ice-cold isolation solution (pH 7.6) containing 250 mM sucrose, 10 mM triethanolamine, PhosSTOP, and cOmplete Mini tablets (Roche Diagnostics A/S, Mannheim, Germany). After centrifugation at 10,000× *g* for 5 min at 4 °C, proteins were extracted using Laemmli sample buffer containing 15 mg/mL DTT. For immunohistochemistry, archived mouse kidney sections were labelled using previously described procedures [23]. A Leica TCS SL confocal microscope (Leica, Mannheim, Germany) with an HCX PL APO ×63 oil objective lens (Leica, Mannheim, Germany) (numerical aperture: 1.40) was used for obtaining images. The brightness of all the images presented here were digitally adjusted.

### 2.5. Western Blotting

Standard procedures were employed for sample preparations and SDS-PAGE. Proteins were transferred electrophoretically onto PVDF membranes (Bio-Rad, Hercules, CA, USA). Immunoreactivity was detected using enhanced chemiluminescence and signal intensity in specific bands was quantitated using Image Studio Lite Ver. 5.2 (LI-COR, Lincoln, NE, USA).

### 2.6. Kidney Tubule Suspensions

Mouse kidney tubules were isolated using a modification of a previous protocol [24]. Briefly, kidneys from C57bl6/J mice were dissected, the capsules were removed, and kidneys immediately placed into digestion buffer (2 mg/mL collagenase in 140 mM NaCl, 0.4 mM KH_2_PO_4_, 1.6 mM K_2_HPO_4_, 1 mM MgSO4, 10 mM Na-Acetate, 1 mM α-ketoglutarate, 1.3 mM calcium-gluconate, and 30 mM glucose). Kidneys were minced into small pieces and digested at 37 °C for 10 min in a thermomixer (Eppendorf, Hauppauge, NY, USA). Isolated tubules were washed three times with cell culture media (DMEM high glucose media containing 1% penicillin/streptomycin) and divided equally into individual aliquots for further treatments. For dDAVP treatment, isolated tubules were pre-incubated 2 h in cell culture media and incubated further in the same media containing either vehicle or dDAVP (10^−9^ M) for 30 min. Following treatment, proteins were extracted using immunoprecipitation (IP) buffer (20 mM Tris, 135 mM NaCl, 5 mM EDTA, 1% NP40) and were used for co-immunoprecipitation studies.

### 2.7. Immunoprecipitation

Immunoprecipitation was performed as described earlier [3]. Briefly, following treatment with either vehicle or dDAVP, samples were lysed using lysis buffer (20 mM Tris, 135 mM NaCl, pH 7.5, 5 mM EDTA, and 1% Nonidet P-40), sonicated and centrifuged at 10,000× *g* for 10 min at 4 °C. A fraction of the lysate was stored separately for analyzing total AQP2 expression. The remaining lysate was transferred to a spin column containing 20 µL of Protein-A-agarose (Santa Cruz Biotechnology, Santa Cruz, CA, USA) and 1 µL of AQP2 antibody and incubated for 60 min at room temperature with end-over-end mixing. After washing three times with wash buffer (phosphate-buffered saline (PBS) with 1% Triton, pH 7.5), proteins were eluted with Laemmli sample buffer containing 15 mg/mL DTT.

### 2.8. Cell Surface Biotinylation

Cells were cultured on semi-permeable supports with dDAVP for 4 days as described above and apical cell membrane proteins were biotinylated and isolated as previously described [22]. Briefly, following a pre-incubation period of 2 h in the absence of dDAVP, cells were treated with either vehicle or dDAVP for 20 min, and apical plasma membrane proteins were labelled with EZ-link hydrozide-biocytin (2.5 mM) and EZ-link NHS-SS-Biotin (1 mg/mL) (Thermo Scientific, Rockford, IL, USA). Cells were then incubated with quenching solution (50 mM NH_4_Cl in PBS, pH 7.4) for 5 min followed by two washes with coupling buffer (0.1 M sodium phosphate and 0.15 M NaCl, pH 7.2). Cells were lysed using lysis buffer (50 mM Tris, pH 7.5, 140 mM NaCl, 5 mM CaCl_2_, 5 mM MgCl_2_, and 1% Nonidet P-40) followed by sonication and centrifugation at 10,000× *g* for 10 min at 4 °C. A fraction of the lysate was stored separately for analyzing total AQP2 protein expression. The remaining lysate was transferred to a spin column containing NeutrAvidin gel slurry (Thermo Scientific, Rockford, IL, USA) and incubated for 60 min with end-over-end mixing to purify biotinylated proteins. After several washes with PBS, labelled proteins were eluted by incubating with Laemmli sample buffer containing 15 mg/mL DTT.

### 2.9. Cycloheximide Protein Half-Life Studies

Cells were grown on semi-permeable supports with dDAVP for 4 days as described above. After washing in DMEM/F12, cells were incubated in cycloheximide (100 μM) for various time periods at 37 °C. Cells were washed with DMEM/F12, proteins extracted using Laemmli sample buffer containing 15 mg/mL DTT and assessed by western blotting. For calculation of the protein half-life, average band densities for each time point were normalized to control and fitted using nonlinear regression and a one-phase exponential decay equation using GraphPad Prism software (version 8). Data were obtained from three independent experiments, with 3–6 observations for each individual time point. 

### 2.10. RNA Isolation and Quantitative Reverse Transcription PCR (RT-qPCR)

Total RNA was isolated using the Ambion Ribopure kit (Thermo Scientific, Rockford, IL, USA) according to the manufacturer’s protocol. cDNA synthesis was performed using Superscript II (Invitrogen, Carlsbad, CA, USA). Quantitative PCR was performed on a Lightcycler 480 (Roche, Mannheim, Germany) using SYBR Green I Master Taq (Roche Applied Science, Mannheim, Germany), with fluorescence measured at the end of each elongation step to calculate Ct values. Relative quantitation of gene expression was determined using the comparative Ct method. Signals for ribosomal 18S amplified in parallel were used to normalize for differences in the amount of starting cDNA. Primers used were; USP4-F: AGCAAGAATCTGAGGCCTGT, USP4-R: GGGTCTCCATGGTGGTGAAG, AQP2-F: TGGCTGTCAATGCTCTCCAC, AQP2-R: GGAGCAGCCGGTGAAATAGA, 18s-F: AGTTCCAGCACATTTTGCGAG, and 18s-R: TCATCCTCCGTGAGTTCTCCA. 

### 2.11. Deubiquitylation Assay

Cells were grown on semi-permeable supports with dDAVP for 4 days as described above. On the day of the experiment, cells were washed twice with DMEM/F12 and incubated in media containing 25 nM 12-tetradecanoylphorbol-13-acetate (TPA) for 15 min at 37 °C to increase ubiquitylated AQP2 levels [25]. AQP2 was immunoprecipitated [22], and a DUB assay performed directly on the AQP2 bound to the protein-G column. Briefly, the column was washed three times using DUB assay wash buffer (20 mM Tris, 0.5 M NaCl, 1% NP40, 5 mM EDTA, and 0.5% Triton, pH 7.4), and incubated with either 100 nM USP4 or 100 nM USP30 (UBPBio, Aurora, CO, USA) for 4 h at 37 °C. Samples were washed three times using DUB assay wash buffer, extracted using Laemmli sample buffer containing 15 mg/mL DTT and assessed by western blotting. 

### 2.12. Statistics

Data in all graphs are shown as mean ± S.E.M. Individual sample size (n) is shown in figure legends. For comparing two groups of data, a Student’s unpaired *t* test was used. For comparisons of more than two groups, one-way ANOVA followed by the Tukey multiple comparison test was used. Significance was considered *p* < 0.05.

## 3. Results

### 3.1. USP4 Is Expressed in AQP2-Containing Cells and Its Abundance Is Modulated by Vasopressin

**Figure 1:**
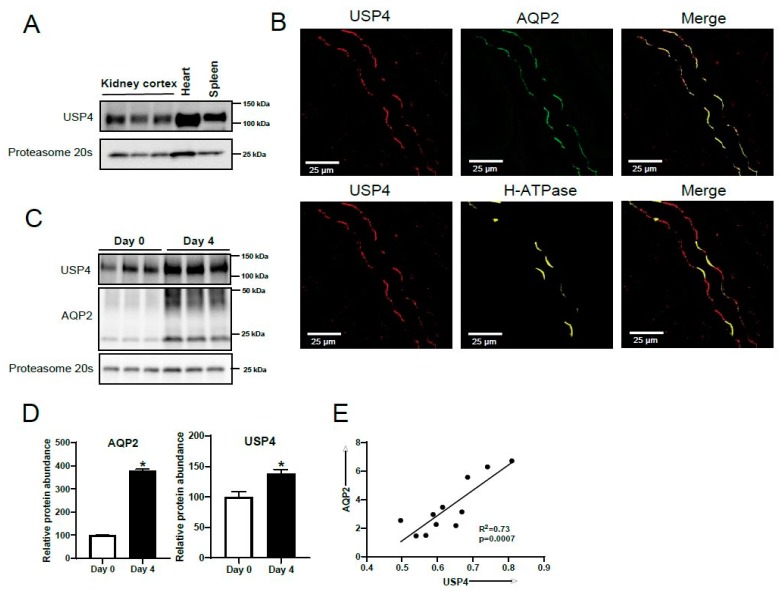
USP4 is expressed in the collecting duct in vivo and its abundance is regulated by vasopressin in vitro. (**A**) Immunoblotting using a USP4-specific antibody demonstrated USP4 to be abundantly expressed in mouse kidney cortex. Each lane represents a sample from an individual mouse, and heart and spleen tissues are positive controls. (**B**) Confocal microscopy images of mouse kidney sections immunolabeled with USP4, the principal cell marker AQP2, and the H+ATPase B1 subunit, an intercalated cell marker. AQP2 and USP4 co-localize at the apical plasma membrane of principal cells. (**C**) Representative immunoblot images of USP4 and AQP2 in mpkCCD14 cells cultured in dDAVP for 4 days. (**D**) Summarized data relative to day 0 (normalized to proteasome 20s). * indicates *p* < 0.05 relative to day 0. (**E**) Linear regression analysis of AQP2 and USP4 protein abundance in mpkCCD14 cells after dDAVP treatment for various periods (0, 24, 48, and 72 h).

Western blot analysis of mouse kidney cortex, heart, and spleen homogenates (positive controls [26,27]) was initially performed to assess if USP4 was found in native kidney tissue, as shown in Figure 1A. A unique protein band of 110 kDa representing USP4 was observed in all samples. To determine if USP4 and AQP2 were localized to the same cells in native kidney, immunolabeling and confocal microscopy was performed on mouse kidney sections using anti-USP4, anti-AQP2, and anti-H+-ATPase B1 subunit antibodies. USP4 was co-localized with AQP2 and not with the H+-ATPase B1 subunit throughout the collecting duct, as shown in Figure 1B. AQP2 and USP4 co-localized predominantly at the apical plasma membrane domain. Our previous study using differential mass spectrometry based proteomics determined that USP4 was abundantly expressed in cultured mpkCCD14 cells (a model of the cortical collecting duct) [9]. To confirm this using an antibody-based approach, and to assess if, like AQP2, USP4 expression is regulated by VP, mpkCCD14 cells were grown on semi-permeable supports and treated with dDAVP (a V2R selective VP analogue) for 4 days. As expected, AQP2 protein levels significantly increased following dDAVP treatment, as shown in Figure 1C,D. Concurrently, USP4 levels also significantly increased, as shown in Figure 1C,D. To assess if the increase in USP4 correlates with the increase in AQP2 after dDAVP treatment, mpkCCD14 cells were treated with dDAVP for various time periods (0, 24, 48, and 72 h), and AQP2 plus USP4 protein abundances were analyzed by western blot. Regression analysis of AQP2 and USP4 abundance in the same sample revealed a linear relationship (R^2^ = 0.735) that deviated significantly from zero (*p* < 0.001), as shown in Figure 1E, suggesting AQP2 levels correlated directly with USP4 abundance following dDAVP treatment.

### 3.2. USP4 Interacts with AQP2 in Cultured Kidney Epithelial Cells and In Vivo

**Figure 2:**
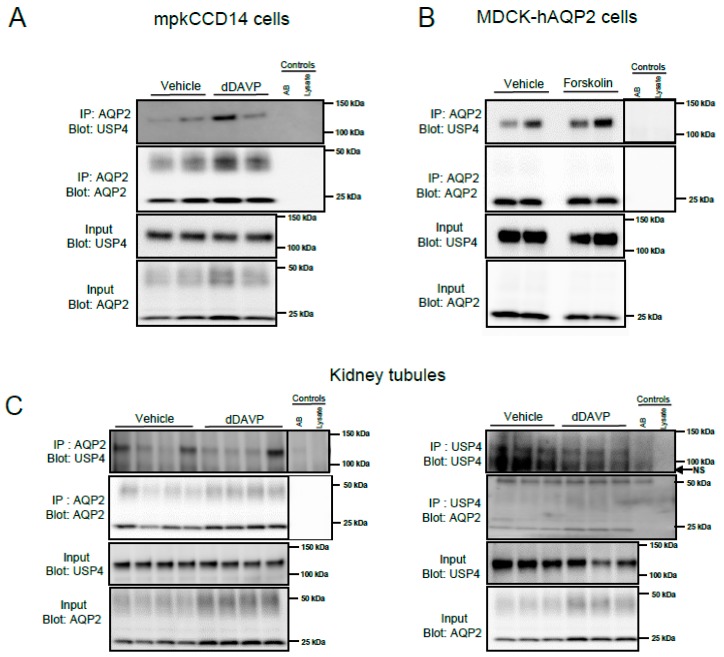
AQP2 and USP4 interact in vitro and in vivo. For studies in cultured mpkCCD14 cells, cells were grown in the presence of dDAVP for 4 days to induce AQP2 expression. On the day of the experiment, cells were washed with DMEM/F12 and incubated in DMEM/F12 for 2 h at 37 °C. Subsequently, cells were treated with either vehicle or dDAVP for 20 min at 37 °C prior to immunoprecipitation (IP) using an anti-AQP2 antibody. For studies in MDCK-hAQP2 cells, on the day of the experiment, cells were washed with DMEM media and subsequently treated with either vehicle or forskolin for 20 min prior to IP using anti-AQP2 antibody. (**A**) Representative immunoblots of USP4 and AQP2 interaction in mpkCCD14 cells. (**B**) Representative immunoblots of USP4 and AQP2 interaction in hAQP2-MDCK cells. (**C**) Representative immunoblots of USP4 and AQP2 in samples immunoprecipitated from mouse kidney tubules acutely treated with either vehicle or dDAVP (30 min) using anti-AQP2 or anti-USP4 antibodies. Each lane represents an individual protein sample. All samples were run on the same membrane and had the same exposure time. Breaks in the images are to show the immunoprecipitation controls (antibody (AB) alone or lysate alone) next to the samples. NS = non-specific band.

To investigate if USP4 interacts with AQP2, co-immunoprecipitation studies were initially performed on lysates from cultured cells. Two different cell lines were utilized, mpkCCD14 cells that have endogenous expression of both USP4 and AQP2 and MDCK-hAQP2 cells [28] that have endogenous expression of USP4 alongside stably transfected human AQP2. In mpkCCD14 cells, USP4 could be immunoprecipitated with an anti-AQP2 antibody, and prior treatment with dDAVP had no clear effect on the interaction, as shown in Figure 2A. Similar results were obtained in MDCK-hAQP2 cells, as shown in Figure 2B, with an interaction between AQP2 and USP4 observed under control conditions, or after treatment of cells with the adenylate cyclase activator forskolin (MDCK-hAQP2 cells have limited response to VP). To examine if USP4 and AQP2 interact in vivo, co-immunoprecipitation studies were performed on lysates from mouse kidney tubule suspensions treated with vehicle or dDAVP for 30 min. USP4 and AQP2 were detected in samples immunoprecipitated using either anti-AQP2 or anti-USP4, confirming that a USP4 and AQP2 interaction occurs in vivo, as shown in Figure 2C. Similar to the cultured cell experiments, prior stimulation of the tubule suspensions with dDAVP had no clear effect on the USP4-AQP2 interaction, as shown in Figure 2C.

### 3.3. USP4 Deubiquitylates AQP2 In Vitro

**Figure 3:**
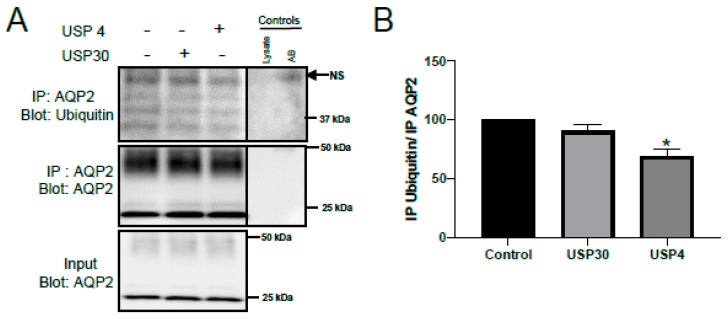
USP4 deubiquitylates AQP2 in vitro. mpkCCD14 cells were grown in the presence of dDAVP for 4 days to induce AQP2 expression. On the day of the experiment, cells were washed twice with DMEM/F12 media and subsequently treated with 25 nM 12-O-tetradecanoylphorbol-13-acetate (TPA) for 15 min at 37 °C. AQP2 was immunoprecipitated (IP) and incubated with either USP4 (100 nM) or USP30 (negative control, 100 nM). (**A**) Representative immunoblots of ubiquitylated and total AQP2 levels following the deubiquitylation assay. + and − indicates presence or absence of the DUB respectively (**B**) Summarized data (n = 3, performed on different days) relative to control. * indicates *p* < 0.05 relative to control conditions. Breaks in the images are to show the immunoprecipitation controls (antibody (AB) alone or lysate alone) next to the samples. NS = non-specific band.

To assess whether USP4 can directly deubiquitylate AQP2, in vitro deubiquitylation assays were performed on AQP2 immunoprecipitated from mpkCCD14 cells. Prior to immunoprecipitation, cells were treated with TPA (25 nM) to increase the levels of ubiquitylated AQP2 [25]. The characteristic pattern [3] of ubiquitylated AQP2 was observed in the immunoprecipitated samples, as shown in Figure 3A. Incubation of immunoprecipitated AQP2 with enzymatically active USP4, but not active USP30, significantly decreased the levels of ubiquitylated AQP2 compared to the control condition, as shown in Figure 3B. This data indicates that a direct and functional interaction between USP4 and AQP2 can occur in vitro to modulate AQP2 ubiquitylation.

### 3.4. Stable USP4 Knockdown in mpkccd14 Cells Alters AQP2 Abundance

**Figure 4:**
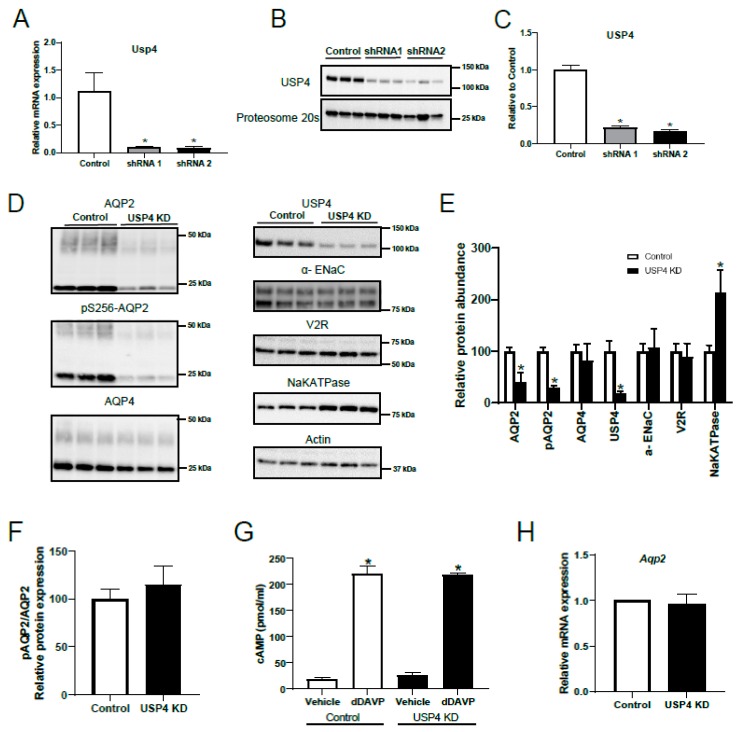
Stable USP4 knockdown in mpkCCD14 cells decreases AQP2 abundance. mpkCCD14 cells with stable shRNA mediated USP4 knockdown or control mpkCCD14 cell lines with scrambled shRNA were generated. mpkCCD14 cell lines were grown in the presence of dDAVP for 4 days to induce AQP2 expression. For studies involving cAMP measurement, on the day of the experiment, cells were washed with DMEM/F12 and incubated in DMEM/F12 for 2 h at 37 °C. Subsequently, cells were treated with either vehicle or dDAVP for 20 min prior to lysis and cAMP measurement. (**A**) Relative mRNA levels of USP4 in control cells or cells expressing two individual USP4 targeting shRNAs (shRNA1 and shRNA2). (**B**) Immunoblotting of USP4 in control, shRNA1, and shRNA2 mpkCCD14 cells demonstrated decreased USP4 protein in knockdown cells. (**C**) Summarized data (n = 3) of USP4 levels in control, shRNA1, and shRNA2 mpkCCD14 cells (normalized to proteasome 20s). * indicates *p* < 0.05 relative to control mpkCCD14 cells. The shRNA1 line was utilized for further studies. (**D**) Representative immunoblots of AQP2, pS256-AQP2, AQP4, USP4, α-ENaC, V2R, Na-K-ATPase α1 subunit and actin protein levels in control and USP4 knockdown mkpCCD14 cells. (**E**) Summarized data relative to control from at least two independent experiments (n = 6). Data were normalized to actin. * indicates *p* < 0.05 relative to control mpkCCD14 cells. (**F**) Relative pS256-AQP2/AQP2 quantification in control and USP4 knockdown mpkCCD14 cells. (**G**) Intracellular cAMP levels in response to dDAVP increased similarly in control and USP4 knockdown mpkCCD14 cells. * indicates *p* < 0.05 relative to vehicle. (**H**) Relative mRNA expression of AQP2 in control and USP4 knockdown mpkCCD14 cells.

To examine the role of USP4 in the regulation of AQP2, several mpkCCD14 cell lines with stable shRNA mediated USP4 knockdown were developed. Control mpkCCD14 cell lines were developed using scrambled shRNA. Following initial characterization of multiple clonal cell lines by examination of cell morphology and the presentation of high TER when grown on semi-permeable supports, two USP4 knockdown mpkCCD14 cell lines were further characterized using RT-qPCR and western blot analysis. Each of these cell lines, developed using shRNAs targeting different regions of the USP4 mRNA sequence, had greatly decreased mRNA levels of *USP4* compared to control mpkCCD14 cells, as shown in Figure 4A. Immunoblotting as shown in Figure 4B indicated that USP4 protein levels in the knockdown cells were reduced by approximately 75% relative to control cells, as shown in Figure 4C. One of the cell lines (shRNA1) was utilized for further experiments (named USP4 KD cells).

Control and USP4 KD cells were grown on semi-permeable supports, treated with dDAVP for 4 days, and the levels of AQP2 or other VP-regulated proteins examined by western blot, as shown in Figure 4D. Relative to control cells, total and Ser256 phosphorylated AQP2 protein levels were significantly reduced in USP4 KD cells, as shown in Figure 4D,E. The changes in the latter were not apparent when phosphorylated AQP2 was normalized to total AQP2 levels, as shown in Figure 4F. As VP signaling in mpkCCD14 cells via the V2R mediates cAMP-dependent increases in AQP2 mRNA and protein levels [22,29], altered V2R activity could explain the decrease in AQP2 observed in USP4 KD cells. However, there was no significant difference in V2R protein levels, as shown in Figure 4D,E, or dDAVP-induced cAMP production between control and USP4 KD cells, as shown in Figure 4G. Furthermore, RT-qPCR analysis determined that the reduced AQP2 protein levels in USP4 KD cells were not a result of altered AQP2 gene transcription, with no significant differences in AQP2 mRNA levels between control and USP4 KD cells, as shown in Figure 4H. Other proteins regulated by VP, such as α-ENaC and AQP4 [30,31], were also not significantly reduced in USP4 KD cells, as shown in Figure 4D,E, whereas Na-K-ATPase α1 subunit protein levels were significantly higher, as shown in Figure 4D,E. Taken together, these data indicate that dDAVP-induced intracellular signaling mediated by the V2R and AQP2 transcription are intact in USP4 KD cells.

### 3.5. Stable USP4 Knockdown in mpkCCD14 Cells Increases AQP2 Ubiquitylation and Reduces AQP2 Membrane Accumulation and Protein Half-Life

**Figure 5:**
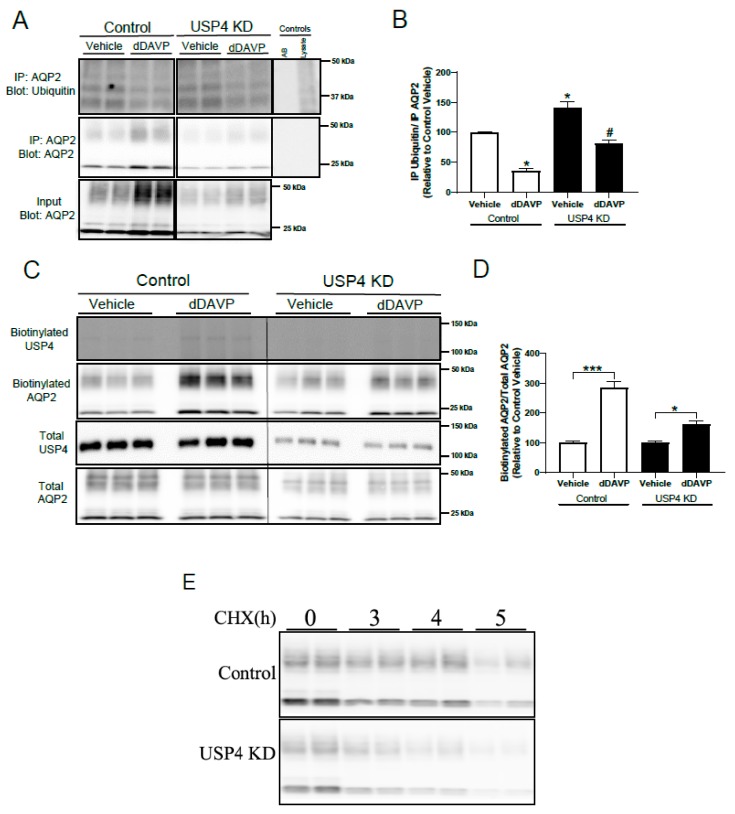
Knockdown of USP4 in mpkCCD14 cells alters AQP2 ubiquitylation levels, apical membrane accumulation, and protein half-life. mpkCCD14 control and USP4KD cells were grown in the presence of dDAVP for 4 days to induce AQP2 expression. For immunoprecipitation (IP) studies, cells were washed with DMEM/F12 and incubated in DMEM/F12 for 2 h at 37 °C. Subsequently, cells were treated with either vehicle or dDAVP for 20 min prior to immunoprecipitation (IP) using an anti-AQP2 antibody. For biotinylation studies, cells were washed twice with DMEM/F12 and subsequently treated with either vehicle or dDAVP for 20 min at 37 °C. For half-life studies, control and USP4 knockdown mpkCCD14 cells were washed with DMEM/F12 and subsequently treated with cycloheximide for different time periods (0, 3, 4, and 5 h). (**A**) Representative immunoblots from immunoprecipitation of ubiquitylated and total AQP2 levels. Breaks in the images are to show the immunoprecipitation controls (antibody (AB) alone or lysate alone) next to the samples. (**B**) Summarized data of ubiquitylated AQP2 to total AQP2 (relative to control vehicle group). Each bar represents mean ± S.E.M obtained from three independent experiments (n = 6). * indicates *p* < 0.05 relative to control conditions and ^#^ indicates *p* < 0.05 relative to vehicle treatment in USP4 knockdown cells. (**C**) Representative immunoblots from biotinylation studies are shown. (**D**) Summarized data of biotinylated AQP2 to total AQP2 (relative to control vehicle group). Each bar represents mean ± SEM obtained from three independent experiments (n = 6). * indicates *p* < 0.05 and *** *p* < 0.005 relative to vehicle treatment. (**E**) Representative immunoblots are shown. Samples were on the same membrane and had the same exposure time.

The previous data indicates that the reduced AQP2 protein levels in USP4 KD cells is likely due to a direct involvement of USP4 in modulating AQP2 ubiquitylation. To examine this, AQP2 was immunoprecipitated from control or USP4 KD mpkCCD cells following vehicle or dDAVP stimulation and the levels of ubiquitin assessed by immunoblotting. Under baseline conditions, ubiquitylated AQP2 levels were significantly higher in USP4 KD cells relative to controls, as shown in Figure 5A,B. dDAVP treatment significantly reduced ubiquitylated AQP2 levels in both control and USP4 KD cells, in line with previous reports [3], however ubiquitylated AQP2 levels remained significantly higher in USP4 KD cells, as shown in Figure 5A,B. Ubiquitylation of AQP2 is involved in modulating AQP2 plasma membrane accumulation and AQP2 degradation [5,9]. To examine the role of USP4 in these mechanisms, AQP2 apical membrane levels were initially examined in control and USP4 KD cells using surface biotinylation, as shown in Figure 5C. In control cells, dDAVP induced a significant increase in AQP2 membrane accumulation in response to dDAVP treatment, as shown in Figure 5D. In USP4 KD cells, although dDAVP increased apical surface AQP2 levels, this was to a lesser extent than in the control cells, as shown in Figure 5D. To explore a role of USP4 in AQP2 degradation, cycloheximide chase studies were performed, as shown in Figure 5E. The calculated AQP2 protein half-life in control mpkCCD14 cells was 5.8 h (95% confidence interval 3.2 to 5.9), which was significantly higher than the 4.2 h (95% confidence interval) in USP4 KD cells. Taken together, these data indicate that loss of USP4 in mpkCCD14 cells increases ubiquitylated AQP2 levels resulting in decreased dDAVP stimulated plasma membrane accumulation and enhanced degradation.

## 4. Discussion

Our previous study detected the deubiquitylating enzyme ubiquitin-specific protease 4 (USP4) in a cell model of the CD (mpkCCD14 cells), where its abundance was approximately 50% greater than in a cell model of the distal convoluted tubule (mpkDCT cells). Based on this, we hypothesized that USP4 had a selective role in the CD to modulate AQP2 function. Our studies highlight that USP4 is abundantly expressed in mouse kidney, where it co-localizes with AQP2 at the apical plasma membrane of collecting duct principal cells. In cell culture, chronic VP treatment increases USP4 levels in direct correlation with increased AQP2 levels. Co-immunoprecipitations of AQP2 with USP4 from a collecting duct cell model (mpkCCD14) and mouse kidney tubules indicated an interaction between AQP2 and USP4. Supporting this, AQP2 and USP4 interacted in vitro, with USP4 being able to directly deubiquitylate AQP2. Taken together, the data suggest that USP4 is a key regulator of AQP2 deubiquitylation.

**Figure 6:**
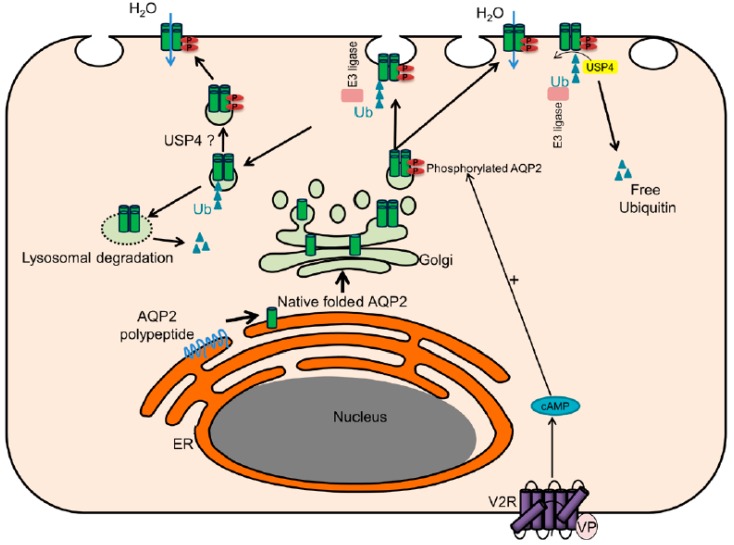
Potential regulation of AQP2 by USP4. Natively folded AQP2 traffics to the Golgi apparatus, is organized into tetramers, and stored in transport vesicles. Following vasopressin (VP) stimulation of the V2R, AQP2 traffics to the plasma membrane (PM) and is phosphorylated at Ser/Thr269, which stimulates AQP2 interaction with USP4. This interaction limits the degree of AQP2 ubiquitylation and allows AQP2 to accumulate on the plasma membrane. Alternatively, following AQP2 internalization, USP4 activity determines whether AQP2 is deubiquitylated and recycled to the PM, or targeted for lysosomal degradation. A number of collecting duct specific E3 ligases may be involved in these processes. Note, the relationship of ubiquitylation and phosphorylation of AQP2 within a tetramer is unknown and the figure does not aim to depict a specific stoichiometry.

Ubiquitylation of AQP2 plays a role in internalization of AQP2 from the apical plasma membrane following VP withdrawal and subsequent lysosomal degradation of AQP2 [5]. In addition, a role of AQP2 ubiquitylation in endoplasmic-reticulum associated degradation (ERAD) via the proteasomal pathway has also been suggested [9]. As the balance between ubiquitylation and deubiquitylation of AQP2 is likely coupled to the regulation of protein levels and activity [32], DUBS likely play an important role in modulating various steps of the AQP2 “lifecycle”. In line with this, knockdown of USP4 in mpkCCD14 cells resulted in increased AQP2 ubiquitylation. This coincided with reduced apical plasma membrane accumulation, reduced protein half-life, and decreased AQP2 protein abundance. Taken together, our observations strongly point to a dual role for USP4 in both limiting the degree of AQP2 endocytosis and stabilizing AQP2, as shown in Figure 6. Two additional mechanisms to be considered in future studies is the role of USP4 and ubiquitylation of AQP2 during autophagy or exosomal secretion of AQP2. In certain acquired forms of nephrogenic diabetes insipidus (NDI) [33], for example hypokalemia- or hypercalcemia-induced NDI [34,35], AQP2 is degraded via autophagy. As substrate ubiquitylation is now considered an important mechanism in autophagy [36], USP4 activity may be inhibited during such forms of NDI to determine selective degradation of AQP2. Alternatively, ubiquitylation of AQP2 may be required for targeting AQP2 to multivesicular bodies and its subsequent secretion via urinary exosomes [37,38,39]. USP4 may play a role modulating the degree of AQP2 sorting to this particular pathway.

Although a number of targets and downstream effects of USP4 have been described e.g., [40,41,42,43], the current literature addresses only a few upstream regulators of USP4 [44,45]. Our study indicated that, at least in cultured cells, chronic VP treatment increases USP4 protein expression. Furthermore, USP4 expression positively correlated with AQP2 expression. These observations contribute to the intricate network of parallel pathways leading from V2R stimulation to the build-up of AQP2 in intracellular storage compartments dependent on the body’s need to conserve water. This build-up of AQP2 depends both on increased synthesis (transcription) [46,47,48] and decreased degradation [49,50]. In respect to degradation, VP-induced phosphorylation of AQP2 at Ser264 has been postulated to direct AQP2 away from lysosomes [51], and phosphorylation of AQP2 at Ser256 and Ser269 (Thr269 in humans) have been associated with limiting lysosomal degradation by increasing AQP2 membrane retention [50]. The predominant apical plasma membrane localization of USP4 coupled to the fact that ubiquitylation of AQP2 occurs on Lys270, allows us to speculate that there is a coupling between the apically associated AQP2-pSer269 [23,52] and USP4 activity. Indeed, a potential cross talk/interdependence of ubiquitylation and phosphorylation of the C-terminus of AQP2 has been addressed previously [3,25,53]. However, dissecting the complex interplay between each of the phosphorylation sites, USP4 interaction domains, and the interplay between the two post-translational modifications (PTMs) is beyond the scope of this study.

A limitation of the current study is that the in vivo role of USP4 is not clear. Mice models lacking USP4 have been developed [26,27], and various mutations in USP4 have been identified in humans e.g., [54]. However, body water homeostasis, renal function, and urinary concentrating capacity in these mice models or patients has not been described. From a clinical perspective, limiting the effects of AQP2 ubiquitylation in respect to degradation of the water channel could be an avenue for the treatment of diseases characterized by water loss due to defective AQP2. For example, some cases of autosomal NDI are due to mutations in AQP2 that result in misfolding and degradation of the protein [55,56]. As this mutant AQP2 may still constitute a partly functional water channel [57], limiting degradation of AQP2 to “rescue” AQP2 expression, or maximizing AQP2 function at the plasma membrane may be of benefit [57,58]. A potential for USP4 acting as such “a rescue factor” are supported by studies demonstrating that USP4 deubiquitylates the cell surface adenosine receptor A_2A_ to enhance its cell surface expression [41]. However, development of drugs against selective DUBs has been limited and currently the focus on DUBs as a therapeutic target focuses exclusively on inhibitors and not activators [32].

In summary, we highlight for the first time specific regulation of AQP2 function by a DUB. Deubiquitylation of AQP2 by USP4 modulates AQP2 membrane accumulation and protein abundance. Our studies suggest an important role of USP4 in modulating the effects of VP in urine concentration and thus enhance our general understanding of body water homeostasis.
